# Prevalence and its associated factors of inadequate dietary diversity among pregnant women attending antenatal care in selected public health facilities in Addis Ababa, Ethiopia

**DOI:** 10.1371/journal.pgph.0005575

**Published:** 2026-01-23

**Authors:** Zinash Getachew Mokonen, Haimanot Teferi Gemaneh

**Affiliations:** 1 Deparment of Psychiatry, Addis Ababa City Administration Health Bureau, Arada Afencho ber Health Center, Addis Ababa, Ethiopia; 2 Department of Medical Laboratory, Addis Ababa City Administration Health Bureau, Bole Dil-frie Health Center, Addis Ababa, Ethiopia; International Institute of Health Management Research - New Delhi, INDIA

## Abstract

Adequate dietary diversity during pregnancy is crucial for the well-being and growth of both the mother and the child. Many pregnant women in Ethiopia, including in Addis Ababa, suffer from poor dietary diversity and associated malnutrition. However, previous studies conducted in Addis Ababa are limited in scope. Therefore, this study assessed inadequate dietary diversity and its associated factors among pregnant women attending antenatal care in selected public health facilities in Addis Ababa, Ethiopia. A facility-based cross-sectional study was carried out among 615 pregnant women in Addis Ababa who were receiving antenatal care at selected public health facilities. A systematic random sampling technique was used to select the study participants. Data were collected using a structured pretested questionnaire. The validated 10 food group dietary diversity questions were used to elicit dietary diversity. This study was conducted in accordance with the Declaration of Helsinki and approved by the Institutional Review Board (IRB) of Menelik II Medical and Health Science Collage (Ref.No.5/38/12/1985). The data were entered on Epi-info version 7.2.1.0 and exported to SPSS version 23.0 for analysis. Logistic regression analysis was used to identify factors associated with low dietary diversity. In general, 55.8% of pregnant women had inadequate dietary diversity. Significant factors included lack of education (AOR = 3.75, 95% CI: 1.96,7.15), low income (AOR = 1.67, 95% CI: 1.02,2.74), lack of nutritional counseling (AOR = 1.68, 95% CI: 1.06,2.68) and household food insecurity (AOR = 5.33,95%CI:2.05,13.81), which were factors associated with inadequate dietary diversity among pregnant women, respectively. The magnitude of inadequate dietary diversity remains high among pregnant women in Addis Ababa. Having low income, being unable to read and write, lacking counseling about dietary diversity, and having household food insecurity were significantly associated with inadequate diversified dietary. Therefore, enhancing women’s education, offering counselling on dietary diversity, and implementing sustainable income-generating and food security interventions activities are crucial.

## Introduction

Dietary diversity, defined as the number of distinct food groups consumed, is a key indicator of diet quality [1].Diversity in diet is necessary to obtain all the nutrients and has been linked to better maternal outcomes, including lower prevalence of micronutrient deficiencies; better pregnancy and birth outcomes; and beneficial effects on child growth [2].

Despite this, low dietary diversity among pregnant women continues to be an important public health problem worldwide, in particular in low and middle-income countries (LMICs). For example, over half of pregnant women in Rwanda and Bangladesh had poor dietary diversity, and low education, limited nutrition knowledge, and household food insecurity were important associated factors [3,4]. Research has suggested that the diets consumed by a large percentage of women during pregnancy in disadvantaged and rural populations are low in diverse, nutrient-rich foods, thereby restricting dietary diversity and increasing the risk of malnutrition [5]. Micronutrient deficiencies during pregnancy are a serious health problem worldwide and contribute to nearly 7.3% of the global burden of disease; multitudes of women are affected by deficiencies in key vitamins and minerals [6].

Studies indicate that women of reproductive age (WRA) consume on average 3.0 to 4.8 food groups, which is below the minimum of five food groups that is recommended. This low diversity results in insufficient consumption of essential micronutrients such as calcium, iron, zinc, vitamin A, thiamine, riboflavin, folate, and vitamin B12. For example, prenatal iron deficiency anemia continues to be a significant issue, seen in 15–20% of pregnant women [7,8].

A remarkable number of pregnant women in Ethiopia could not get a diverse and nutritious diet. National estimates indicate that approximately 53% of pregnant women consume a diet that is not sufficiently diverse, and there is a low pooled estimate of adherence to the minimum diversity window (41%) [9,10]. Similarly, a community-based cross-sectional study found that only 38.4% (95% CI: 33.7–43.2) of pregnant women practiced adequate dietary diversity, with maternal education, meal frequency, home gardening, and household food security identified as key determinants [3]. A 2023 facility-based study revealed that 55.4% of pregnant women had adequate dietary diversity, with urban residence, home gardening, higher meal frequency, and nutrition counselling during ANC visits positively associated [4]. This low diversity in diet can lead to intrauterine growth restriction and risk of having adverse birth outcomes. Insufficient maternal dietary intake is also an issue that will negatively affect both the quality and the quantity of breast milk, an inviolable element during the first 1000 days of life that is necessary for the optimal growth and development of the child [11].

The Ethiopian Ministry of Health has introduced interventions to enhance maternal health with respect to nutrition, via social media, and training for health extension workers. Nutritional counseling is recommended for pregnant women to raise awareness of healthy eating practices [12]**.** However, despite these efforts, only 47% of women report understanding the importance of a varied, balanced diet. This issue is especially concerning in urban areas like Addis Ababa, where rapid urbanization, socio-demographic inequalities, food insecurity, and poverty present additional barriers to dietary diversity [13,14]. Furthermore, studies in Addis Ababa show that only 27% of women have adequate knowledge of maternal nutrition, with 48.4% demonstrating a favorable attitude and only 34.5% reporting healthy eating habits [15,16].

In Addis Ababa, the capital city of Ethiopia, limited research has explored the factors influencing dietary diversity among pregnant women attending antenatal care. This evidence gap has hindered the creation of interventions to target the nutrition needs of this at-risk population. Knowledge of the determinants of maternal dietary diversity is important for appropriate messages to optimize maternal and child health. Diversity of diet during pregnancy is also essential for meeting Sustainable Development Goals, specifically SDG3 [Good Health and Well-being], which seeks to reduce maternal and child mortality, stunting, and malnutrition.

Therefore, this study was going to assess dietary diversity and its associated factors among pregnant women attending antenatal care in selected public health facilities in Addis Ababa, Ethiopia. The findings will help inform interventions to improve maternal nutrition and child health outcomes, guide healthcare providers in promoting better dietary practices, and provide a foundation for future research in this area.

## Materials and methods

### Ethics statement

This study was conducted in accordance with the Declaration of Helsinki and approved by the Institutional Review Board (IRB) of Menelik II Medical and Health Science Collage (Ref.No.5/38/12/1985). A formal letter of permission was sent to Addis Ababa city administration health bureau, and permission was obtained from each sub-city Health office and the respective health facilities Directors. The interviewers were ensuring that the respondent’s understood the purpose of the study, study procedures, and benefits before the consent was given. The study participants (adults (≥18 years)) were informed that they had the right to participate or decline to participate in the study. The participant’s confidentiality was assured, and all information collected was treated confidentially by avoiding their name and other personal identifying information. The interview was taking place after informed written consent was obtained from each participant.

### Study setting, period, and, study design

A facility-based cross-sectional study was conducted from January 15 to February 15, 2025, in selected public health facilities of Addis Ababa. Addis Ababa is the capital city of Ethiopia, located in the central highlands at an elevation of 7,500 feet above sea level. Today, it is the largest city in Ethiopia and the second-most populous city in Africa after Lagos, Nigeria, with a population of approximately 4 million people as of the 2024 population projection, of whom approximately 2.1 million are females (52.5%) and 1.9 million are males (47.5%) [17]. According to the information from the Addis Ababa City Health Bureau, in Addis Ababa city, there are 96 public health facilities and the number of pregnant mothers attending antenatal care is 122,716 (Addis Ababa City Health Bureau Report, personal communication, August, 2024).

### Population and eligibility

All pregnant women attending antenatal care in Addis Ababa health facilities were the Source population. All pregnant women attending antenatal care at selected health facilities in Addis Ababa who met the inclusion criteria were the study population. All pregnant women chosen from the study population to participate in the study were study subjects. Pregnant women attending antenatal care at the selected public health facilities during the study period were included in the study. Pregnant women with chronic diseases such as cancer and diabetes, and those who were unable to communicate due to illness, and those in the first trimester (because early pregnancy is often associated with nausea and reduced food intake, which may not reflect the usual dietary pattern) were excluded from the study.

### Sample size determination and sampling

A single population proportion formula was used to calculate a sample size, assuming a 95% confidence level for which Z = 1.96, 5% margin of error, 10% non-response rate, and 51.6% proportion of inadequate dietary diversity reported from a previous study in Addis Ababa, Ethiopia [13].The formulae (n = Z² (p q)/ d²) substituted n= (1. 96)² (0.516) (1-0.516)/ (0.05) ² = 384. Then, after a 10% non-response rate was added, the sample size became 423, and because multistage sampling was used, the sample size was multiplied by a moderate design effect of 1.5 [18], the final sample size was 635. Sample size for the second objective was determined using Epidemiology Information (Epi info) statistical calculation considering 80% power and 1:1 ratio based on a study conducted in Addis Ababa, Ethiopia [13].Since the sample size calculated for the first objective could accommodate the second objective, 635 was selected.

**Fig 1:**
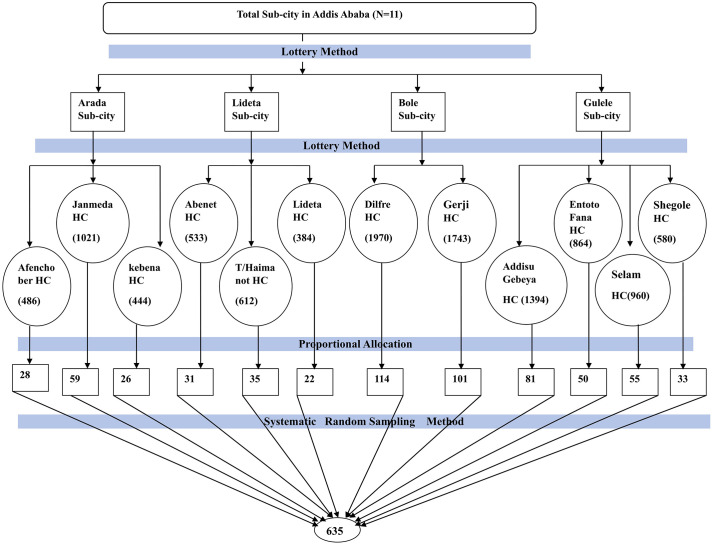
Schematic presentation of sampling procedure for the study on inadequate dietary Diversity and its associated risk factors among pregnant women attending antenatal care in Addis Ababa, Ethiopia, 2025.

The study facilities were selected from public health institutions in Addis Ababa. The selection of participants was carried out using a multistage sampling method. The first stage was the selection of sub-cities: of the 11 sub-cities in Addis Ababa, four (Arada, Bole, Lideta, and Gulele Sub-city) of them were selected by a simple random sampling technique (lottery method). The second stage was the selection of institutions from identified sub-cities: accordingly, 12 Health centers were selected by lottery method from their respective sub-cities; namely, Arada sub-city (Afenoch Ber, Janmeda, and Kebena Health centers), Bole sub-city (Dilfre, and Gerji Health center), Lideta (Abenet, T/haimanot, and Lidet Health centers), and Gulele (Addisu Gebeya, Entoto Fana, Selam, and Shegole Health centers). The sample size (635) was proportionally allocated to the selected public health facilities (Fig 1), and participants in each facility were selected by using a systematic sampling technique after calculating the sampling interval (K) for each facility. The sampling interval K was determined by dividing the average number of pregnant women who antenatal care follow-up by the desired sample size (K = N/n = 10991/635 = 17). The first sample was selected by lottery method between 1 and 17, and then every 17^th^ interval values were selected to get required sample size from each public health facility. In order to avoid double-counting, pregnant women coming for follow-up were asked if they had been interviewed for this study in previous days and excluded from the interview if they had been.

### Study variables and operational definition

#### Dependent variable.

Dietary Diversity for women (MDD-W)

#### Independent variables.

Age, marital status, mother’s educational status, husband educational status, average income, household food security, mother’s occupational status, husband’s occupational status, number of pregnancies, Family size, number of ANC visit, number of meals/days, additional diet, illness during pregnancy, diet-related knowledge, attitude, and dietary counseling.

#### Operational definition.

**Adequate dietary diversity** is defined as the consumption of five or more food groups out of the ten food groups recommended by the Food and Agriculture Organization (FAO); otherwise, less than the consumption of five food groups is considered **inadequate dietary diversity** [19]

Pregnant women with **poor nutritional knowledge** were those who score less than or equal to mean score of knowledge assessment questions. On the other hand, pregnant women with **good nutritional knowledge** were those who score above the mean score of knowledge assessment questions [20].

Pregnant women were considered to have a **positive attitude** if she scored more than the mean score out of the questions. Conversely, if she scored below or equal to the mean score, she was considered to have a **negative attitude** [20].

### Data collection method and procedures

Data were collected using a pre-tested structured interviewer-administered questionnaire, comprised of five parts. Part I consists of questions related to socio-demographic and maternal health-related factors. Parts II consist of knowledge-related questions. Part III comprises attitude-related questions. Part IV contains household food insecurity, and Part V involves the Dietary Diversity score question.The questionnaires were adapted from different related studies [13,21–23].

Dietary diversity was evaluated by relying on the pregnant mother’s recall of food items consumed in the 24 h preceding the survey. A total of ten food groups (i.e., 1.Grains, white roots and tubers, and plantains, 2.Pulses (beans, peas, and lentils), 3.Nuts and seeds, 4.dark green leafy vegetables, 5.other vitamin A-rich) vegetables and fruits, 6.other vegetables, 7.other fruits, 8.Dairy, 9.Meat, poultry and fish,10 egg) will be used [19]. Each group was assigned a score of “1” point if they were consuming any of the foods in each subgroup at least once in the past 24 h and “0” points if they didn’t consume the food at all. The dietary diversity score was calculated by adding the number of foods categories consumed for 24 h. The data collectors ask a series of standard probing questions to help the respondent recall all foods and beverages consumed the previous day and night, and also probe for main ingredients in mixed dishes. In particular, the recall period lasts for 24 hours, starting from the moment the respondent woke up the day before and ending with the day and night. The recall was “open” because the interviewer didn’t read predefined foods/groups to the respondent. On a predetermined list, each item of food or drink that the respondent named was marked, underlined, or ticked. Foods not already included on the predefined list were classified by the interviewer into an existing predefined food group.

The questionnaire included ten questions designed to evaluate a pregnant woman’s knowledge of dietary intake and recommendations during pregnancy, and the questionnaires were adapted from the previous literature and conceptualized it to local situations [24]. A knowledge score was calculated for each participant based on the number of questions correctly answered in the knowledge assessment section. Each correct response was coded as ‘’1”and an incorrect response as “0.” Attitudes towards dietary diversity were assessed by asking nine attitude questions, each with three options. Pregnant women receive a score based on their responses: 3 points for agreed, 2 points for neutral, and 1 point for disagreed, following a Likert scale. Then, the aggregate attitude score was determined for each pregnant woman by summing up the scores across the nine attitudes related questions. The total score was then categorized based on mean values into “good” or “poor” knowledge and “positive or” negative” attitude [20].

### Data quality assurance

To ensure the quality of the data, the questionnaire was initially developed in English and then translated into the local languages (Amharic) and back-translated into English by independent language experts to ensure its consistency. The questionnaire was pretested on 5%(N = 32) of the total estimated sample size in Aware health center, which had similar characteristics to the study subjects in a different adjacent setting, and the questionnaire was modified based on the findings of the pre-test, such as identifying and replacing ambiguous words. Twelve BSc nurses as data collectors were recruited, and a two-day training session was provided on the objectives of the study, methodology of the data collection, and ethical issues to minimize interviewers’ bias. To ensure the accuracy of the data, the principal investigator closely followed the data collectors daily for the successful completion of the questionnaire and took timely action in case of any deviations.

### Data analysis

First, each questionnaire was cleaned and checked for completeness. Then, the Data were coded and entered into the computer using Epi Info 7.2.1.0 and exported to the SPSS program version 21.0 for further analysis. Frequency tables and charts were used to display the data. The outcome variable was recorded as dichotomous outcomes: inadequate DDS was coded as “1” and adequate as “0”. Bivariate logistic regression analysis was done to explore the crude association between different predictor variables and the Dietary diversity score. To control for possible confounding factors and to identify factors that are independently associated with the outcome variable, multivariable logistic regression analysis was performed for those variables with p value of less than 0.25 in the bivariable analysis. The Adjusted Odds Ratio (AOR) with a 95% confidence interval was used to indicate the strength of association, and a P-value of <0.05 is used to declare the statistical significance in the multivariable analysis. The Goodness of fit for the final regression models was checked by the Hosmer-Lemeshow (0.495).

## Results

### Sociodemographic characteristics

The study involved 615 pregnant women, with a 97% response rate. The average age of pregnant women was 30.2 ± 5.3 years. Among pregnant women, the age group of 24–34 was 414(67.3%), which is over half of the total, and 96.4% were married. More than one third (37.2%) of the study participants and 45.9% of their husbands were with college and above, respectively. Of the participants in the study, one-fourth were housewives (25.4%), while more than one-third (45.4%) of their husbands were employed as Privates. The study revealed that over half (52.2%) of pregnant women with a monthly income below 10,000 [Table 1]

**Table 1:** Socio-economic and demographic chrematistics of pregnant women attending antenatal care in selected public health facilities in Addis Ababa, Ethiopia/2025(N = 615).

Variables	Frequency	(%)
**Age**		
< 24	60	9.8
24–34	414	67.3
≥ 35	141	22.9
**Marital Status**		
Married	593	96.4
Single	19	3.1
Widowed/Divorced	3	0.5
**Pregnant Women’s Educational Level**		
Unable to read and write	82	13.3
Reading and writing	78	12.7
Primary	137	22.3
Secondary	89	14.5
College and above	229	37.2
**Pregnant Women’s Occupational Status**		
Housewife	156	25.4
Private employer	224	36.4
Government employer	140	22.8
Merchant	42	6.8
Student	21	3.4
Daily laborer	32	5.2
**Husband’s Educational Level**		
Unable to read and write	29	4.9
Reading and writing	74	12.5
Primary	65	10.9
Secondary	153	25.8
College and above	272	45.9
**Husband’s Occupational Status**		
Private employer	269	45.4
Government employer	195	32.9
Merchant	97	16.4
Daily laborer	32	5.3
**Average Household Income (ETB/Month)**		
< 10,000	321	52.2
10,000–20,000	195	31.7
> 20,000	99	16.1
**Family Size**		
< 4	510	82.9
≥ 4	105	17.1

Additionally, 65.7% of participants were multigravida, and 55.8% had fourth and above ANC visits. A significant proportion of the sample 510(82.9%), had fewer than five family members, and 78% received nutritional advice from a healthcare provider. A majority of pregnant women (53.2%) had over 3 meals per day, while a further two-third, 420(68.3%), included extra food during pregnancy, and less than one-fourth,132(21.5%), were sick in the last four weeks. A significant proportion of households, 363 (59%), were found to have food insecurity, and 41% were food secure. The majority of pregnant women, 372(60.5%) had a good nutritional knowledge, while more than two-third, 417(67.8%), had a positive attitude [Table 2].

**Table 2:** Obstetric and general health related information of pregnant women attending antenatal care in selected public health facilities in Addis Ababa, Ethiopia/2025(N = 615).

Variables	Frequency	(%)
**Number of ANC visit**		
Two	110	17.9
Three	162	26.3
Four and above	343	55.8
**Gravidity**		
Primigravida	211	34.3
Multigravida	404	65.7
**Nutrition Education/Advice**		
Yes	493	80.2
No	122	19.8
**Eating frequency during the day**		
3	288	46.8
3+	327	53.2
**Additional diet during pregnancy**		
Yes	420	68.3
No	195	31.7
**Illness in the last four weeks**		
Yes	132	21.5
No	483	78.5
**Nutritional Knowledge**		
Good	372	60.5
Poor	243	39.5
**Attitude**		
Positive	417	67.8
Negative	198	32.2
**Household Food Security level**		
Food secured	252	41.0
Mild food insecure	181	29.4
Moderate food insecure	149	24.2
Severe food insecure	33	5.4

### Prevalence of Inadequate dietary diversity among pregnant women

**Fig 2:**
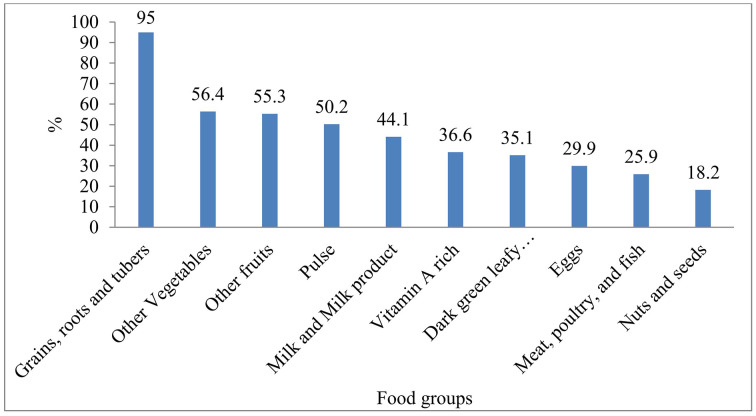
Food group consumption pattern of pregnant mother attending antenatal care in selected public health facilities in Addis Ababa, Ethiopia, 2025.

**Fig 3:**
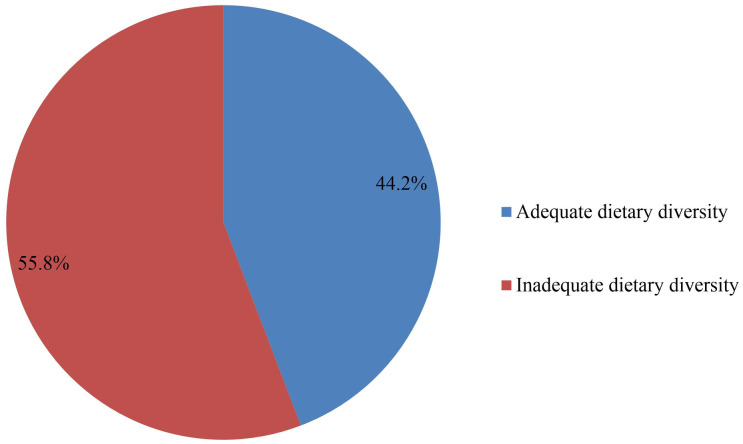
Prevalence of Inadequate dietary diversity among pregnant women attending antenatal care in selected public health facilities in Addis Ababa, Ethiopia, 2025.

In pregnant women, the most commonly consumed foods (95%) were grains, roots, and tubers (teffe, wheat, corn/maize, and barley), while nuts (18.2%) and meat (25.9%) were the least frequently eaten [Fig 2]. The prevalence of inadequate dietary diversity among pregnant women in the 24 hours before the survey was 55.8% (95% CI: 51.88%–59.72%) [Fig 3].

### Factors associated with inadequate dietary diversity of pregnant women

Bivariable logistic regression analysis revealed that out of eighteen explanatory variables, only seven variables, such as respondent educational status, family income, number of pregnancies, additional diet during pregnancy, nutritional knowledge,nutritional advice during pregnancy, and food insecurity, had a p-value <0.25 with pregnant women’s inadequate dietary diversity practice, whereas age, marital status, respondent occupation, husband educational and occupational status, family size, number of ANC visits, meal frequency, illness,and attitudes were variables with a p-value _≥_ 0.25 and excluded from further analysis. Those variables with a p-value < 0.25 during bi-variable analysis were entered into multivariate logistic regression in order to rule out the effect of confounding variables. As a result, four of the contributing factors, such as the educational status of the pregnant mother, family income, nutritional advice during pregnancy, and food insecurity, remained to be significantly and independently associated with pregnant women’s inadequate dietary diversity (p-value <0.05) [Table 3].

**Table 3:** Bivariable and multivariable logistic regression analysis showing associated factors for inadequate dietary diversity among pregnant women attending antenatal care in selected public health facilities in Addis Ababa, Ethiopia, 2025.

Variables	Inadequate DD		COR(95% CI)	AOR(95% CI)	P-value
	YesN(%)	NoN(%)			
**Educational level**					
Unable to read & write	66 (19.2)	16 (5.9)	4.30(2.36,7.84)	3.75(1.96,7.15)	0.0001*
Read and write	44 (12.8)	34 (12.5)	1.35 (0.81, 2.25)	1.29 (0.74, 2.24)	0.363
Primary	76 (22.2)	61 (22.4)	1.30 (0.85, 1.98)	1.15 (0.73, 1.81)	0.556
Secondary	38 (11.1)	51 (18.8)	1.07 (0.64, 1.80)	1.00 (0.58, 1.73)	0.989
College and above	119(34.7)	110(40.4)	1	1	—
**Average income**					
< 10,000	195(56.9)	125(46.0)	1.95 (1.24, 3.08)	1.67 (1.02, 2.74)	0.042*
10,000–20,000	104(30.3)	92 (33.8)	1.41 (0.87, 2.30)	1.63 (0.97, 2.73)	0.066
> 20,000	44 (12.8)	55 (20.2)	1	1	—
**No of pregnancies**					
Multigravida	237(69.1)	167(61.4)	1.41 (1.01, 1.97)	1.39 (0.96, 1.99)	0.080
Primigravida	106(30.9)	105(38.6)	1	1	—
**Additional diet**					
No	120(35.0)	75 (27.6)	1.41 (1.00, 1.20)	1.03 (0.70, 1.52)	0.891
Yes	223(65.0)	197(72.4)	1	1	—
**Nutritional knowledge**					
Poor	148(43.1)	95 (34.9)	1.41 (1.02, 1.96)	1.22 (0.84, 1.77)	0.306
Good	195(56.9)	177(65.1)	1	1	—
**Nutrition Education**					
No	80 (23.3)	42 (15.4)	1.67 (1.10, 2.52)	1.68 (1.06, 2.68)	0.028*
Yes	263(76.7)	230(84.6)	1	1	—
**Food Security level**					
Severe food insecurity	25 (7.3)	6 (2.2)	5.04(2.01,12.66)	5.33(2.05,13.81)	0.001*
Moderate food insecurity	95 (27.7)	42 (15.4)	2.74 (1.78, 4.21)	2.88 (1.83, 4.52)	0.000*
Mild food insecurity	94 (27.4)	68 (25.0)	1.67 (1.13, 2.50)	1.78 (1.19, 2.67)	0.005*
Food secure	129(37.6)	156(57.4)	1	1	—

COR: Crudes odds ratio, AOR: adjusted odds ratio, CI: Confidence interval, *: p < 0.05

In this study we observed that pregnant women who were unable to read and write were nearly four times more likely to possess inadequate diet diversity than those who had a college or higher education level (AOR = 3.75, CI: 1.96,7.15 The likelihood of experiencing inadequate dietary diversity was 1.67 times higher in families with monthly incomes below 0,000 ETB compared to those who had an estimated monthly income of greater than 20,000 ETB (AOR = 1.67, CI: 1.02,2.74). This study found that pregnant women who received no nutritional advice or dietary diversity information from their health care provider were 1.68 times less diverse than those who received information about their diet (AOR = 1.68, CI: 1.06,2.68). Similarly, pregnant women from mild, moderate, and severe food-insecure households were 1.78, 2.88, and 5.33 times more likely to have poor dietary diversity than those from food-secure households.

## Discussion

This study estimated the prevalence of inadequate dietary diversity among pregnant women who visited antenatal care. The study revealed that more than half, 55.8% (95% CI: 51.9%–59.7%) of pregnant women had poor dietary diversity. This finding aligns with the national pooled rate of poor dietary diversity in Ethiopia (53%) [25], Addis Ababa (51.6%) [13], Bale (55.2%) [23], Dire Dawa (55%) [21], and Gojjam(53.2%) [22]. Still, lower than in Hiwot Fana (65%) [26], Injibara (61.8) [27], and Shashemane (74.6)[28]. Outside Ethiopia, similar patterns have been documented. In Rwanda, 55.9% of pregnant women had inadequate dietary diversity [4]; in Tanzania, only 28% achieved the minimum dietary diversity [29]; and in Bangladesh, about 59% of pregnant women were found to have inadequate dietary diversity [3]. These similarities across different low- and middle-income countries suggest that inadequate dietary diversity during pregnancy remains a widespread public-health concern linked to common socioeconomic and structural factors.

Furthermore, this study revealed that a pregnant woman’s dietary diversity level had a strong relationship with her level of education. Therefore, those mothers who cannot read and write had 3.75 times poorer dietary diversity practice compared to those mothers a college-level or higher level of education.This result is consistent with a study conducted in Hiwot Fana Specialized University Hospital(AOR = 3.01) [26],Dire Dawa(AOR = 3.8)[21],Injibara (AOR = 3.4)[27], Bellessa (AOR = 4.0) [30], Shashemane (AOR = 2.13) [28], and Addis Ababa (AOR = 1.7)[13] and other countries such as Nepal [31] and Ghana [32], which reported that educated women are more likely to understand the importance of diversified diets and have better access to nutrition information. The explanation for this can be due to those mothers with a level of college and above being likely to access information about nutritional needs and have a better understanding of mass media-delivered educational messages. Moreover, a pregnant woman with education has more consciousness about how to use available resources for enhancing quality of diet. Thus, with increasing educational level, it can be expected to raise the level of accomplishing enough dietary diversity intake [33].

The analysis found that pregnant women with lower monthly income (<10,000) had 1.67 times a higher probability of poor dietary diversity compared to their counterparts. This coincides with a survey conducted in Dire Dawa(AOR = 4.4) [21],Bellessa(AOR = 4.46) [30],Addis Ababa (AOR = 2.5) [13], and Shashemane (AOR = 2.24) [28]. Similar associations between income and dietary diversity have been reported among pregnant women in Tanzania [29] and Bangladesh [3], suggesting that household economic capacity remains a key driver of maternal diet quality. High-income women are likely to have better buying power, which can allow them to purchase a more varied diet, although not easily available at home. Pregnant women from low-income backgrounds are likely to have difficulty paying for diversified food.

This study also indicated that pregnant mothers not receiving advice/education regarding the necessity for dietary diversity were 1.68 times more likely to have improper dietary diversity when compared to those who received dietary diversity information. This finding concurs with a studies from Bale(AOR = 5.26) [23],Dire Dawa (AOR = 2.2) [21], Bellessa(AOR = 2.15) [30], and a systematic review (AOR = 2.75) [25] done in Ethiopia. The provision of information about a diet with diversity is directly related to dietary diversity among pregnant women. Pregnant women receiving guidance/education about dietary diversity have a better understanding concerning a balanced diet leading to optimal health status and a favorable pregnancy outcome.

Pregnant women had a likelihood of having poor dietary diversity by a factor of 1.78, 2.88, and 5.33 times when they experienced mild, moderate, and severe household food insecurity, respectively, compared with having household food security. This was also confirmed by another study done in Bellessa(AOR = 2.63) [30], Mettu Karl Referral Hospital(AOR = 3.66) [34],A Systematic Review and Meta-Analysis in Ethiopia(AOR = 2.18) [25],and Western Ethiopia(AOR = 2.63) [35].This shows that food-secure households are likely to eat a variety of foods and have access to multiple food groups. Contrary to this, during food insecurity, pregnant women are likely to use a variety of coping strategies, including eating less frequently, eating a restricted diet, and eating inadequate quantities of both macronutrients and micronutrients, leading to poor dietary diversity.

## Limitation of the study

Dietary data were obtained from participants’ self-reported food intake in the last 24 hours, which can be subject to recall bias. So, data collectors use probing questions to help respondents recall all foods, beverages, and main ingredients consumed the previous day and night. The 24-hour dietary recall gives a one-day picture of dietary intake, which might not be reflective of participants’ usual or habitual dietary habits. Pregnant women might have exaggerated intakes of nutritious foods or might have underestimated intakes of unhealthy foods because of a socially desirable response. This could have affected the estimate.

## Conclusion and Recommendations

This study revealed the high prevalence of inadequate dietary diversity among pregnant women attending antenatal care in public health facilities of Addis Ababa. Low educational status, limited household income, lack of dietary counseling during ANC visits, and household food insecurity were significantly associated with inadequate dietary diversity. Strengthening nutritional counseling services within ANC programs, improving women’s education and economic empowerment, and enhancing household food security interventions are essential to improve maternal dietary practices. Health professionals and program planners should integrate targeted nutritional education and behavioral-change communication during antenatal care. Future research is recommended to employ longitudinal or intervention-based designs to explore causal relationships and evaluate the effectiveness of tailored nutritional interventions in improving dietary diversity among pregnant women.

## Supporting information

S1 Data Dataset used for the analysis of of Inadequate Dietary Diversity among Pregnant Women Attending Antenatal Care in Selected Public Health Facilities in Addis Ababa, Ethiopia. (SAV)
